# Preliminary analysis of salivary microbiome and their potential roles in oral lichen planus

**DOI:** 10.1038/srep22943

**Published:** 2016-03-10

**Authors:** Kun Wang, Wenxin Lu, Qichao Tu, Yichen Ge, Jinzhi He, Yu Zhou, Yaping Gou, Joy D Van Nostrand, Yujia Qin, Jiyao Li, Jizhong Zhou, Yan Li, Liying Xiao, Xuedong Zhou

**Affiliations:** 1 State Key Laboratory of Oral Diseases, West China Hospital of Stomatology, Sichuan University, Chengdu, China; 2 Department of Marine Sciences, Zhejiang University, Zhejiang, China; 3 Institute for Environmental Genomics, Department of Microbiology and Plant Biology, University of Oklahoma, Norman, OK, USA

## Abstract

Several studies have explored the origin and development mechanism of oral lichen planus (OLP) with limited attention to the role of bacteria in the progression of this common oral disease. Here we utilized MiSeq sequencing of 16S rRNA gene amplicons to identify complex oral microbiota associated with OLP from saliva samples of two subtypes (reticular and erosive) of OLP patients and healthy controls. Our analyses indicated that the overall structure of the salivary microbiome was not significantly affected by disease status. However, we did observe evident variations in abundance for several taxonomic groups in OLP. *Porphyromonas* and *Solobacterium* showed significantly higher relative abundances, whereas *Haemophilus*, *Corynebacterium*, *Cellulosimicrobium* and *Campylobacter* showed lower abundances in OLP patients, as compared with healthy controls. In addition, we explored specific microbial co-occurrence patterns in OLP, and revealed significantly fewer linkers of *Streptococcus* comprising species in erosive OLP. Furthermore, the disease severity and immune dysregulation were also genus-associated, including with *Porphyromonas* that correlated to disease scores and salivary levels of interleukin (IL)-17 and IL-23. Overall, this study provides a general description of oral microbiome in OLP, and it will be useful for further investigation of their potential roles in the initiation and immune modulation of OLP.

Lichen planus is a common chronic mucocutaneous inflammatory disease affecting skin, oral, genital mucosa, scalp and nails. The prevalence of oral lichen planus (OLP) ranges from 0.1–4% in the general population, and females between 30 and 60 are more vulnerable to this disease^1^. Lesion manifestations can often persist for years alternating between periods of quiescence and exacerbation^2^. Intraorally, OLP presents as white striations, papules, plaques, mucosal atrophy, erosions or blisters, mainly affecting the buccal mucosa, tongue, gingiva and lips^3^. Among those clinical phenotypes, the reticular form is the most prevalent lesion characterized by the presence of Wickham striae and usually asymptomatic. Whereas the erosive type, although less frequent, always causes different degrees of discomfort and soreness, it may need ongoing concern due to its malignant potential^4^.

Despite of numerous literatures appraising the OLP origin and development mechanism, its aetiology remains uncertain, and the pathogenesis of this disease still needs more investigations. Several internal and external factors have been implicated, such as immunodeficiencies and microbial infection^5^. Several lines of evidences have suggested that immune dysregulation and complex cytokine network played important roles in the exacerbation and perpetuation of OLP^6^,^7^. For instance, a high proportion of the novel CD4^+^ T helper cells subset-Th17 cells were detected in OLP lesions^8^. And interleukin (IL)-17 and IL-23, the newly discovered Th17-associated cytokines, were found to be important proinflammatory factors involved in OLP^9^,^10^. In terms of infection with oral microorganisms, several viruses have been linked with OLP^11^,^12^,^13^,^14^,^15^. Besides that, Lundstrom *et al.* demonstrated *Candida* infection in 37–50% of OLP patients^16^. An association between *Helicobacter pylori* and OLP has been shown but remains controversial^17^,^18^,^19^. While not directly involved in the etiology of OLP, all of these factors could influence the composition of oral microbiota, which might in turn modulate immunity and thereby affect the progression of this disease. An early report noticed that intensive oral hygiene could provide improvement of some patients with atrophic and ulcerative gingival lichen planus^20^. Backman and Jontell demonstrated that the treatment of chlorhexidine could considerably relieve the symptoms of lichenoid lesions^21^. A recent study found significantly higher counts of *Staphylococcus haemolyticus* and *Streptococcus agalactiae* in OLP lesions^22^. These findings propelled the further exploration of the links between oral bacteria and OLP. Previously, we evaluated the differences in genetic profiling of salivary microbial community between OLP and matched healthy controls by means of polymerase chain reaction-based denaturing gradient gel electrophoresis^10^. To investigate the microbiota associated with OLP more extensively, in this study, we further applied with Illumina MiSeq sequencing to identify the microbiome in saliva samples from two subtypes (reticular and erosive) of OLP patients and healthy controls.

In the present study, we aimed to (1) characterize the overall structure of oral microbiota in saliva of OLP patients; (2) seek for OLP-associated microorganisms; (3) elucidate the changes in microbial interactions of OLP patients; and (4) identify key microbial populations associated with disease and immune status. Our results indicated that even though the microbial community composition has not dramatically altered, some abundant genera still have changed obviously. The interactions of microbial-microbial and microbial-immune might play a key role in OLP pathogenesis directly or indirectly.

## Results

### The phylogenetic structure of OLP microbial communities showed a greater variety and less specificity

The demographic and clinical characteristics of all subjects are shown in Table 1. Statistical analysis failed to reveal significant group differences in age and gender (P = 0.127 and P = 0.815 respectively).

To investigate the changes in structure and composition of salivary microbial communities of OLP, 55 samples were sequenced with Illumina MiSeq technology. After preprocessing, 2,706,496 high quality sequences with average length of 253 bp were obtained in this study, with an average of 49209 sequences per sample. From these sequences, 13 known phyla and 418 genera were identified, and a total of 8717 OTUs were detected, of which 4006 were singletons (detected in only one sample) and were excluded from further statistical analysis. Good’s estimator of coverage was 99.85%, indicating that the 16S rRNA sequences identified in this study likely represent the majority of bacterial sequences present in saliva samples. The sequencing data for details are available in Supplementary Table S1.

**Figure 1:**
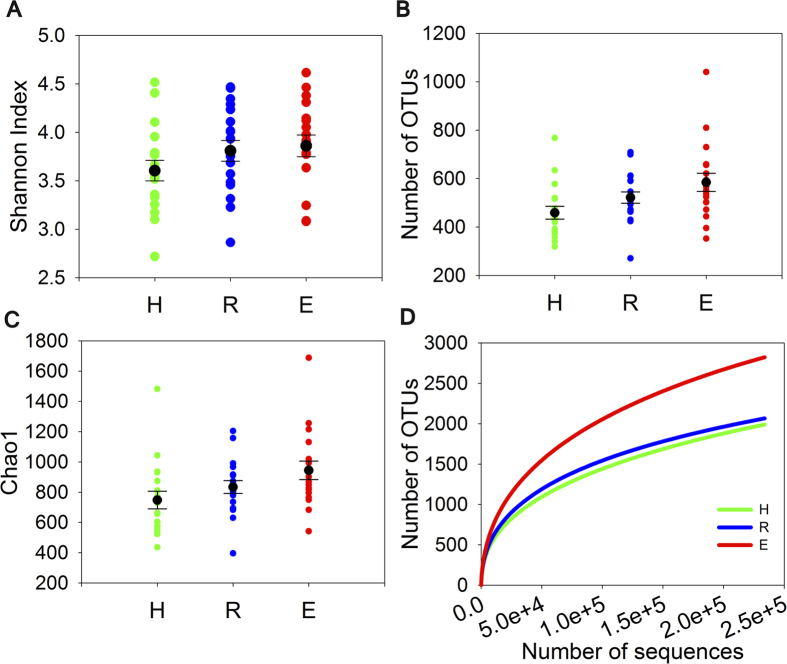
Alpha Diversity analysis for healthy controls (H), reticular OLP (R) and erosive OLP (E). No significant differences between healthy and diseased samples were observed for various alpha diversity measures (P > 0.05). The scatter plots were shown with black spots and horizontal lines representing the mean and standard deviation, respectively. (**A**) Shannon Index. (**B**) Number of Operational taxonomic units (OTUs) generated with a minimum pairwise identity of 97%. (**C**) Chao1 richness. (**D**) Rarefaction curves of the number of OTUs in each sample.

Principal coordinates analysis (PCoA) plot based on Bray-Curtis distances indicated no obvious separation among healthy subjects, reticular OLP and erosive OLP (Supplementary Figure S1). The dissimilarity tests among groups did not show marked differences as well (P > 0.05, Table 2). Oral microbial diversity was similar between OLP patients and healthy controls (P > 0.05, Fig. 1A–C, Supplementary Table S1). There was a slightly higher richness detected in erosive OLP than that in other two groups, but no statistical significance was found. The rarefaction analysis showed a total of 1989, 2054, and 2823 OTUs (at 239,000 sequencing depth) in healthy controls, reticular OLP and erosive OLP respectively, indicating that more phylotypes would likely be detected in erosive OLP than other groups after exploring larger number of sequences (Fig. 1D).

**Figure 2:**
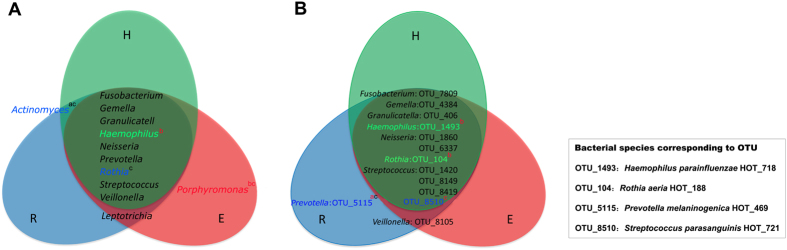
The core microbiota of saliva samples in three experimental groups. The model included the genera (above 1% of relative abundance) and OTUs (above 0.3% of relative abundance) in healthy controls (green), reticular OLP (blue) and erosive OLP (red), which were found in at least 50% of subjects. Colored labels indicate genera and OTUs having a greater relative abundance in healthy controls (green), reticular OLP (blue), and erosive OLP (red). Species corresponding to statistically different OTUs are listed in the square box. Statistical differences were calculated by Student’s *t* test. a: H vs R; b: H vs E; c: R vs E. Superscript letters in red indicate P < 0.05, and those in black indicate P < 0.1.

To investigate the existence of a disease-specific microbiome in saliva of OLP patients, we evaluated the genera and OTUs which were found in at least 50% of subjects with a mean relative abundance of >1% and >0.3% respectively. As a result, the core genera are shown in Fig. 2A. In this range, *Porphyromonas* was only found in erosive OLP patients, while *Leptotrichia* was detected in reticular and erosive OLP patients. Although *Actinomyces* was detected in both reticular OLP and healthy controls, this genus was indeed more abundant in reticular OLP. Additionally, using the BLAST method against the HOMD to assign the sequences to bacterial species, we found that *Prevotella melaninogenica* (OTU5115) was unique in reticular OLP and *Veillonella parvula* (OTU8105) dominated in reticular and erosive OLP when compared with healthy controls (Fig. 2B).

### Changes of oral microbiota at the phylum, genus and OTU levels in OLP patients

**Figure 3:**
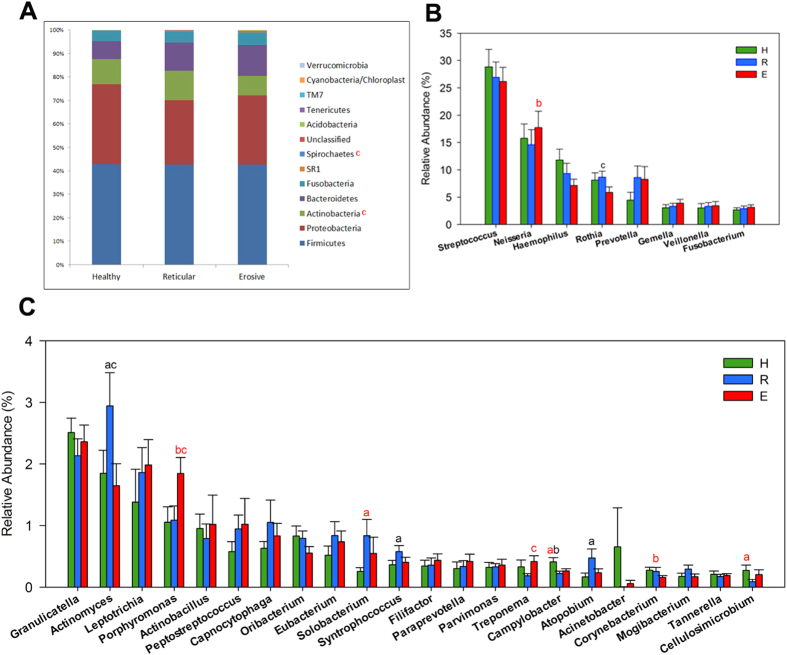
Relative abundance of bacterial phyla and predominant genera (>0.1%) among healthy controls (H), reticular OLP (R) and erosive OLP (E). (**A**) Phylum level. Compared with reticular OLP, Spirochaetes was more abundant, and yet Actinobacteria was less abundant in erosive OLP. (**B**) Comparison among top 8 abundant genera. (**C**) Comparison among top 9 to 30 abundant genera. a: H vs R; b: H vs E; c: R vs E; Superscript letters in red indicate P < 0.05, and those in black indicate P < 0.1.

The salivary microbial community composition of patients with OLP and healthy individuals were analyzed at different taxonomic levels. The overall microbial community composition of three groups at the phylum level was shown in Fig. 3A. As the histogram displayed, the most predominant phylum of all groups was Firmicutes, followed by Proteobacteria, Actinobacteria, Bacteroidetes and Fusobacteria. The phylum Spirochaetes was more abundant in erosive OLP over reticular OLP (P = 0.02), and yet Actinobacteria showed more abundance in reticular OLP over erosive OLP (P = 0.01). Meanwhile, the abundance of some genera differed significantly. Only the genera with average relative abundance >0.1% were demonstrated in Fig. 3B,C. As compared with healthy controls, the relative abundance of genus *Porphyromonas* was increased in erosive OLP, and *Solobacterium* was more abundant in reticular OLP. In contrast, *Haemophilus*, *Corynebacterium, Cellulosimicrobium* and *Campylobacter* showed significantly overabundance in healthy group than diseased groups. Nonetheless, few species had significantly different abundance levels among three groups. *Haemophilus parainfluenzae* (OTU1493), *Rothia aeria* (OTU104) and *Streptococcus parasanguinis* (OTU8510) were less abundant in erosive OLP, while *Prevotella melaninogenica* (OTU5115) had significantly higher levels in reticular OLP (Fig. 2B).

### Co-occurrence patterns of salivary microbiota in OLP

**Figure 4:**
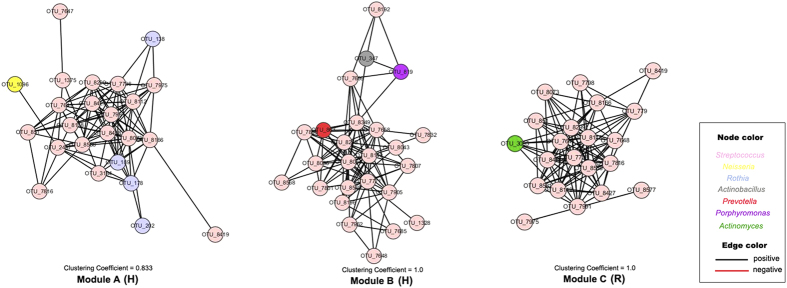
Co-occurrence networks of connected microbial clusters (modules) of *Streptococcus* comprising species. Each node represents an OTU colored by the genus-level phylotypes, and each edge represents a significant co-occurrence relationship colored by the sign of the association (red negative, black positive). Two highly connected microbial clusters (Module A,B) in healthy controls (H), and one module (Module C) in reticular OLP (R) were observed in the networks.

The goal of network inference is to identify combinations of microorganisms demonstrating significant co-occurrence or mutual exclusion patterns across samples and to predict ecological relationships. By analyzing and then visualizing the spatial Pearson’s correlations between the bacterial species detected from saliva samples, decreased network complexity, as measured by average connectivity, could be observed for microbial co-occurrence networks in reticular and erosive OLP groups. For example, we captured 1094 associations among 282 nodes in healthy group, 1185 associations among 335 nodes in reticular OLP group, and 985 associations among 324 nodes in erosive OLP group at the OTU level (Supplementary Figure S2). In the network, pairwise relationships were represented by edges connecting two nodes. The nodes with the most numerous linkers, which were defined as hub nodes, were different among three groups. The hub species were *Atopobium sp.* (OTU7853 co-occurring with 29 OTUs) in healthy controls, *Prevotella melaninogenica* (OTU661 co-occurring with 31 OTUs) in reticular OLP, and *Solobacterium moorei* (OTU771 co-occurring with 24 OTUs) in erosive OLP, respectively. In addition, to further explore the biological significance of co-occurrence relationships of oral microbiota, we identified highly connected microbial clusters of the genus *Streptococcus* in the network (Fig. 4). Interestingly, clear reduction of *Streptococcus* co-occurrence network patterns could be observed from healthy individuals to patients with reticular and erosive OLP. In healthy controls, the genus *Streptococcus* comprising species were clustered into two modules (module A and B) associated with OTU7975 and OTU7832 respectively. In reticular OLP, there was only one module (module C) of *Streptococcus* associated with OTU8419, but corresponding nodes did not form any modules in erosive OLP. Also, we found that the OTUs representing the genus *Streptococcus* had significantly fewer linkers in erosive OLP than those in reticular OLP and healthy controls (P < 0.05 and P < 0.001, respectively). This indicated that *Streptococcus* might have played an important role in the development of OLP.

**Figure 5:**
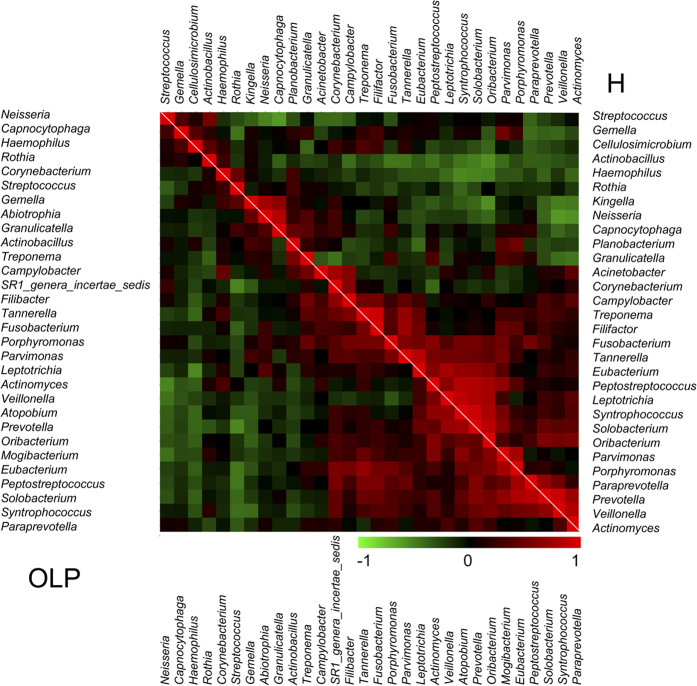
Co-occurrence and co-exclusion analysis of the bacterial genera. The analysis was calculated by Pearson correlations among the top 30 abundant bacterial genera. The co-occurrence of the abundant genera in OLP and healthy samples was shown on the left and right, respectively. The correlation values ranged from −1.00 (green) to 1.00 (red).

We further employed the co-occurrence and co-exclusion analysis of the top 30 abundant bacterial genera in detail. Two subtypes of OLP patients were combined into the OLP group for this analysis. Out of 26 abundant genera which were shared between the diseased and healthy groups, we found significant changes in the ecologically competitive interactions of these bacteria (Fig. 5). In healthy controls, *Treponema* and *Filifactor* were the most positively correlated (ρ = 0.945), whereas *Capnocytophage* and *Streptococcus* were the most negatively correlated (ρ = −0.601). But in OLP, the most positively correlated genera were *Solobacterium* and *Peptostreptococcus* (ρ = 0.839), while *Streptococcus* and *Prevotella* were the most negatively correlated (ρ = −0.540). Besides, Opponent co-occurrence patterns were observed for several microbial genera in diseased and healthy status. For example, *Treponema* and *Actinobacillus* were correlated negatively (ρ = −0.249) in healthy group, but their correlation converted to be positive (ρ = 0.564) in OLP group. The same pattern was also observed for the genera *Actinomyces* and *Granulicatella.*

### Genera associated with the clinical parameters

**Figure 6:**
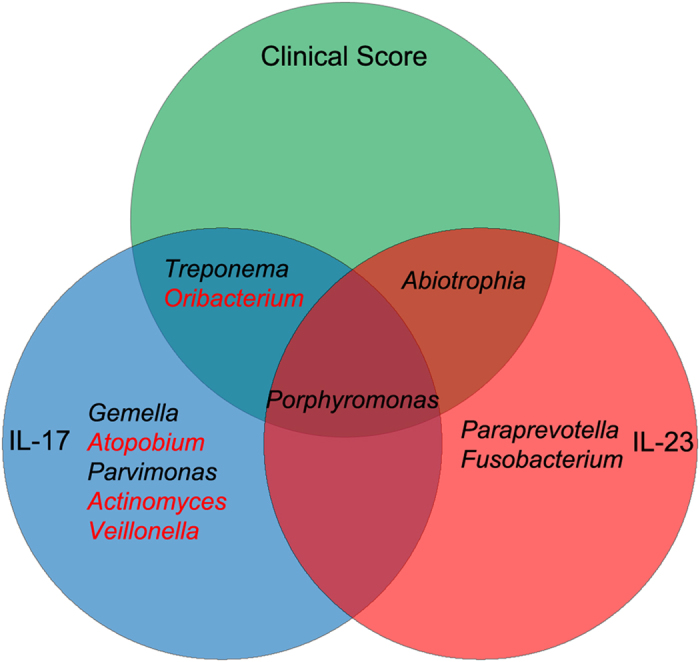
The bacterial genera associated with disease scores and proinflammatory cytokines. The model displayed all genera associated with each of the three clinical parameters of OLP (P < 0.05). The genera name in red were correlated negatively with clinical data, and those in black were correlated positively with clinical data.

The concentrations of IL-17 and IL-23 were measured by means of enzyme-linked immunosorbent assay (ELISA) and the results were shown in Supplementary Table S2. To further investigate the association between oral microorganisms and the clinical data, Pearson correlation coefficients were calculated (Supplementary Table S3). A significant correlation was observed between some detected genera and clinical parameters, including the clinical scores and salivary levels of IL-17 and IL-23 (Fig. 6). First, the genera including *Treponema, Porphyromonas* and *Abiotrophia* were positively correlated with clinical scores, whereas *Oribacterium* was negatively correlated with clinical scores. Second, it was observed that many genera were correlated with immunologic factors involved in the inflammation response in OLP. For example, *Treponema*, *Porphyromonas*, *Gemella* and *Parvimonas* showed significantly positive correlations with the salivary levels of IL-17, whereas *Abiotrophia*, *Porphyromonas*, *Paraprevotella* and *Fusobacterium* were found to be positively correlated with the salivary levels of IL-23. Finally, some of these genera associated with clinical data had significant correlations with more than one parameter, such as *Porphyromonas*, which was correlated positively with the clinical scores and salivary levels of IL-17 and IL-23 at the meantime.

## Discussion

The role of the viral infection in OLP has already come into notice. However, there is a paucity of knowledge regarding the role of bacteria in the progression of this common oral disease. In the present study, we described the complexity of microbial communities in OLP, and our results uncovered crucial yet striking salivary microbiomic characteristics associated with disease. The microbial community structure displayed a greater variety and less specificity in OLP patients. And the samples from erosive OLP displayed even higher phylogenetic diversity than healthy controls, as its rarefaction curves did not yet reach a plateau with current sequencing. These results are not in agreement with findings of a recent study^10^. This discrepancy may be due to differences in sequencing methods, bioinformatic analyzing methods and the number of subjects included in different studies. Our results implicated the association between changes in the microbial enrichment and OLP.

Among three groups, five major phyla constituted the predominant salivary microbiome (that is, Firmicutes, Proteobacteria, Actinobacteria, Bacteroidetes and Fusobacteria), consistent with previous surveys^23^,^24^. Significant differences of oral microbiome at the phylum and genus levels were identified between OLP patients and healthy individuals. The relative abundances of phylum Actinobacteria, Spirochaetes and its genus *Treponema* differed between two subtypes of OLP patients, and these microbial taxa may be associated with disease classification of OLP. *Porphyromonas* was discovered to be dominant in erosive OLP, which was also considered to be the core genus in periodontitis^25^,^26^. As the implementation of our sample collection had excluded those subjects with periodontal inflammation or other oral diseases, we could assume that the abundance shift of *Porphyromonas* had something to do with OLP state. Our findings supported the results of a previous study, in which higher periodontopathogens’ percentages were detected in OLP groups than those in the non-OLP groups^27^. This periodontopathogenic genus with high percentage may also play an important role in the etiology of OLP.

Moreover, analysis at the OTU level confirmed the disease-associated species, which increased in relative abundance and prevalence, including *Veillonella parvula* and *Prevotella melaninogenica.* Also, higher counts of *Veillonella parvula* was examined at sites with evidence of OLP lesions using DNA-DNA hybridization in a previous study^22^. *Prevotella* species, the Gram-negative rod-shaped anaerobes, were also implicated involved in many other oral disease, such as endodontic disease^28^, periodontal infection^29^ and dental caries^30^. *Prevotella melaninogenica,* as the hub species with significantly higher abundance, could be regarded as the core bacteria in reticular OLP. Findings reported here underlined the potential role of this reticular OLP-associated species played in the disease. While in this study, *Haemophilus parainfluenzae*, *Rothia aeria* and *Streptococcus parasanguinis* were less abundant in erosive OLP compared with healthy controls. Indeed, in a recent study, Said *et al.*^31^ reported that the salivary microbiome of patients with inflammatory bowel disease comprised higher proportions of *Veillonella* and *Prevotella* and lower proportions of *Haemophilus* and *Streptococcus* than those in healthy controls, and they indicated that the dysbiotic genera *Veillonella* and *Prevotella* could elicit an inflammatory response in mucosa. Although the abundance of the genera *Veillonella* and *Prevotella* were not significantly different between OLP patients and healthy individuals, they might be important for the pathogenesis of OLP at the species level. However, further investigation is required to clarify the role of these dysbiotic oral microbiota in the progression of this disease.

Oral microorganisms rarely survive in isolation, but instead coexist in complex ecology with various symbiotic relationships^32^. Previous studies identified that individual microbial interactions were not only essential for community stability but also involved in dysbiosis and overgrowth of pathogens linked to disease^24^,^33^. In our study, the co-occurrence analysis revealed significant changes in the interactions of salivary microbiota in OLP. For example, in diseased group, the suspected bacteria *Porphyromonas* with higher abundance turned to be correlated significantly with *Eubacterium*, which was also found to be involved in periodontitis^25^ and peri-implantitis^34^. These shifts of the symbiotic and antagonistic relationships suggested the existence of microbial dysbiosis in OLP. Groups of densely connected molecules usually have an important biological significance in the interaction networks^35^. Our network analyses showed significantly decreased interactions of *Streptococcus* comprising species, also supporting the imbalance of oral microbiota in OLP. Equally, the decrease of Streptococcaceae comprising species associated with oral health was observed in ulcerated sites of recurrent aphthous stomatitis (RAS) patients^36^. Potential effect of Streptococci-based products in controlling oral inflammatory conditions has been suggested recently^37^. Our findings may not exclude a role for these products in preventing the exacerbation of OLP.

There is an increasing body of evidence suggesting that perturbations to the structure of complex commensal communities can modulate innate and adaptive immune response and lead to the development of immune mediated diseases, with inflammation arising upon the reduction of the proportion of symbiont microbiota or the increase in the number of pathogenic microbiota^36^,^38^. With the identification of Th17 cell, the importance for its defense against extracellular and intracellular bacterial infection has been demonstrated in a mass of researches^39^,^40^. Previously, we demonstrated the potential association between oral microbial diversity and levels of IL-17 in saliva of OLP patients^10^. In this study, we further explored 7 genera that correlated positively to the Th17-associated cytokines (IL-17 and IL-23), and these bacteria might participate in dysimmunity and inflammation maintaining in OLP. Most notably, out of these genera, the anaerobic bacteria *Porphyromonas* positively correlated to three clinical parameters deserves more attractions. As one of the most frequently encountered species in human, *Porphyromonas gingivalis* has recently been demonstrated on its ability to subvert host immunity and promote dysbiosis of oral microbiota^41^. Besides, *Porphyromonas gingivalis* was also indicated to enhance the Th17 cell responses for the development of atherosclerosis^42^. Together with detection of significantly higher abundance of *Porphyromonas* in erosive OLP, our findings highlight the urgency of the further research on the role of this bacteria played in OLP. Additionally, we postulated that some microbiota colonized in saliva of OLP patients might be associated with the severity of disease. The genera *Treponema*, *Porphyromonas* and *Abiotrophia* showed significantly positive correlations with the clinical scores, suggesting these bacteria might be involved in the exacerbation of OLP and their proportion may signify the degree of severity of this disease. *Treponema, Porphyromonas, Parvimonas* and *Fusobacterium* have recently been reported to be the core microbiota in periodontitis^25^. The potential roles these periodontopathogenic microorganisms play in the progression of OLP was also reported recently^27^, and our results agreed with those of the previous study. In addition, higher levels of *Treponema* and *Porphyromonas* in erosive OLP compared to those in reticular OLP and healthy individuals also supported their association with disease severity. While the genus *Abiotrophia*, which is often associated with culture-negative infective endocarditis^43^, was found to be correlated with disease severity and IL-23 levels in our study. We assume that these relevant microorganisms, together or separately, might play a direct role in the initiation of OLP or act as triggers of immune response directly or indirectly following the formation of OLP lesions.

In conclusion, we preliminarily identified the microbial taxa associated with OLP and delineated different features of salivary microbiome between OLP and healthy individuals, providing a comprehensive cross-sectional description of oral microbiota in OLP. Our study afforded deep insight into the dysbiosis in the microbial community of OLP, suggesting a potential microbial role in progression of this disease. Also, Our findings could provide the basis for further exploring the bacteria suspected of being associated with OLP in the future studies.

## Methods

### Subject recruitment and sample collection

Subjects with reticular OLP (n = 19) and erosive OLP (n = 18) aged between 22–62 years were selected during follow-up clinical examinations from the West China Hospital of Stomatology, Sichuan University, and the OLP patients were diagnosed in accordance with the clinical classification and definition of OLP by the World Health Organization^44^. The degree of severity of the disease was based on the clinical scores evaluated by a semiquantitative scoring system^45^ in line with the site, area and presence of lesions. Additionally, 18 healthy volunteers who met the requirements of having matched gender and age with the OLP patients were recruited as controls. All subjects were willing to consent to the clinical examination and saliva sampling. They had neither detectable periodontal inflammation and visible caries lesions, nor any severe systemic disorders and smoking history. Moreover, they had not received treatment for OLP or taken prescription drugs at least two months before sampling. This study was approved by the local ethics committee of West China Hospital of Stomatology, Sichuan University. Informed consent was obtained from all study participants. The methods were carried out in accordance with the approved guidelines.

According to the standard techniques described by Navazesh^46^, about 5 ml spontaneous, whole unstimulated saliva (WUS) was collected in a sterile DNA-free conical tube from each subject between 8:00~11:00 am. Subjects were refrained from drinking and eating for at least 2 h before sampling. All samples were transported to laboratory on ice within 2 h, and stored at −80 °C before further processing.

### Cytokines assay

The levels of IL-17 and IL-23 in saliva were detected as described previously^10^.

### DNA extraction

Genomic DNA was extracted from saliva samples using the QIAamp DNA micro Kit (Qiagen, Valencia, CA, USA) according to the manufacturer’s instructions with minor modifications by adding a lysozyme (3 mg ml^−1^, 1.5 h) treatment step. Quality of the extracts was evaluated by the absorbance ratios at A260/A280 and A260/A230 using spectrophotometry (NanoDrop 1000, Thermo Scientific Inc, Wilmington, DE, USA). Only DNA samples with A260/A280 >1.7 and A260/A230 >1.8 were used in downstream experiments. The final concentrations of DNA were quantified using the Pico-Green kit (Invitrogen, Carlsbad, CA, USA). DNA samples were frozen at −20 °C until further analysis.

### Illumina sequencing and bioinformatics analysis of 16S rRNA gene amplicons

Bacterial 16S rRNA genes from each sample were amplified using F515 (primer seq) and R806 (primer seq) primers targeting V4 hypervariable regions and then sequenced with the Illumina MiSeq technology at the Institute for Environmental Genomics, University of Oklahoma (Norman, OK, USA). The MiSeq 500 cycles 250 × 2 V2 kit was used for sequencing 16S rRNA V4 on MiSeq machine (Illumina, San Diego, CA). Data preprocessing and OTU clustering was performed as described previously^25^. Taxonomic assignment was carried out using the RDP classifier with confidence cutoff value of 50%^47^.

The sequence data were further analyzed with the following methods: (A) Principal coordinate analysis (PCoA) was used to determine the degree of dissimilarity between pairs of bacterial communities using Bray-Curtis distance method. Three different nonparametric analyses for multivariate data implemented by the “vegan” package in R were also used for community functional structure comparison, including multiresponse permutation procedure (MRPP), nonparametric multivariate analysis of variance (adonis) using distance matrices and analysis of similarities (ANOSIM). (B) Alpha-diversity indices were estimated by the Shannon diversity index, the number of observed OTUs, and Chao1 species richness estimates. Rarefaction curves were applied to calculate the species richness based on the bacterial OTUs and were then compared among three groups by the mothur package^48^. (C) Microbial-microbial co-occurrence networks were constructed by the MENA pipeline^49^. A Pearson correlation cutoff of 0.74 was determined by random matrix theory approach by observing a transition point of nearest-neighbor spacing distribution of eigenvalues from Gaussian to Poisson distribution, which are two universal extreme distributions. In such networks, OTUs were represented by network nodes while correlations were transformed into links between them. The co-occurrence networks were then visualized using Cytoscape 3.2.0 with a force-directed algorithm^50^, and network topological parameters were computed using NetworkAnalyzer^51^. (D) Co-occurrence patterns of the 30 most abundant taxonomic groups across samples were explored by calculating the Pearson correlation coefficients. Results were clustered and visualized by the MeV package^52^.

Student’s *t-*test was used to compare microbial diversity and relative abundances of each bacterial taxon. Pearson correlations were used for the analyses of co-occurrence and bacterial associations with the clinical data.

## Additional Information

Accession codes: The data are available at the NCBI Sequence Read Archive (SRA) under accession no. SRP067603 (http://www.ncbi.nlm.nih.gov/sra). 

**How to cite this article**: Wang, K. *et al.* Preliminary analysis of salivary microbiome and their potential roles in oral lichen planus. *Sci. Rep.* **6**, 22943; doi: 10.1038/srep22943 (2016).

## Supplementary Material

Supplementary Information

## Figures and Tables

**Table 1:** Demographics and clinical parameters of all subjects.

Characteristics	Healthy subjects (n = 18)	Reticular OLP patients (n = 19)	Erosive OLP patients (n = 18)
Age (Mean ± SD)	39.72 ± 11.02	43.42 ± 9.56	46.72 ± 9.80
Male/Female	5/13	7/12	5/13
Disease severity score	0	1~3	4~5
Body mass index (Mean ± SD)	20.22 ± 1.37	21.14 ± 1.12	20.56 ± 1.78
Diabetes	0	0	0
Cigarette smoking	0	0	0
Alcohol drinking	0	0	0
No. of teeth (Mean ± SD)	29.78 ± 1.77	28.43 ± 2.25	28.78 ± 2.58
Plaque index (Mean ± SD)	1.32 ± 0.43	1.22 ± 0.51	1.40 ± 0.47
Gingival index (Mean ± SD)	1.08 ± 0.54	1.29 ± 0.45	1.23 ± 0.52
Bleeding on probing (+/−)	1/18	2/18	1/17
Probing depth/mm (Mean ± SD)	2.01 ± 0.71	2.21 ± 0.40	2.17 ± 0.56
Clinical attachment loss/mm (Mean ± SD)	0.67 ± 0.50	0.73 ± 0.54	0.75 ± 0.43

**Table 2:** Comparison of the overall microbial community structure using three nonparametric statistical methods.

Groups	MRPP	P-value	Adonis	P-value	ANOSIM	P-value
H vs R	0.511	0.252	0.036	0.209	0.011	0.294
H vs E	0.513	0.158	0.040	0.155	0.021	0.210
R vs E	0.520	0.302	0.032	0.263	0.008	0.326

(H, healthy control; R, reticular OLP; E, erosive OLP).
