# Supplementary material for: Preliminary analysis of salivary microbiome and their potential roles in oral lichen planus
Source: Sci Rep. 2016 Mar 10;6:22943. doi: 10.1038/srep22943 (PMC4785528; doi:10.1038/srep22943)
Supplement: Supplementary Information [file srep22943-s1.doc]

**Preliminary analysis of salivary microbiome and their potential roles in oral lichen planus**

Kun Wang^1, 4^, Wenxin Lu^1, 4^, Qichao Tu^2, 3^, Yichen Ge^1^, Jinzhi He^1^, Yu Zhou^1^, Yaping Gou^1^, Joy D Van Nostrand^3^, Yujia Qin^3^, Jiyao Li^1^, Jizhong Zhou^3^, Yan Li^1,*^, Liying Xiao^1,*^, Xuedong Zhou^1,*^

^1^State Key Laboratory of Oral Diseases, West China Hospital of Stomatology, Sichuan University, Chengdu, China;

^2^Department of Marine Sciences, Zhejiang University, Zhejiang, China

^3^Institute for Environmental Genomics, Department of Microbiology and Plant Biology, University of Oklahoma, Norman, OK, USA

^4^These two authors contributed equally to this work.

*Correspondence: Yan Li, E-mail: [feifeiliyan@163.com](mailto:feifeiliyan@163.com), Liying Xiao, E-mail: klobme@163.com, Xuedong Zhou, zhouxd@scu.edu.cn, State Key Laboratory of Oral Diseases, West China Hospital of Stomatology, Sichuan University, Chengdu 610041, China.

**Supplementary Figures**

**Supplementary Figure S1. Principal coordinate analysis (PCoA) plot based on Bray-Curtis distance method.** Each symbol represents a sample. No distinct group clustering was apparent within any sample groups.

**Supplementary Figure S2. Network inference for the complex microbial relationships in healthy controls (H), reticular OLP (R) and erosive OLP (E).** Each node represented an OTU, and each edge represented a significant pairwise association (red negative, black positive) between them. Significant relationships were detected with the hypergeometric distribution. The square boxes contains highly connected microbial clusters formed by OTUs representing the genus *Streptococcus* (see more details in Figure 4).

**Supplementary Figure S1**

**
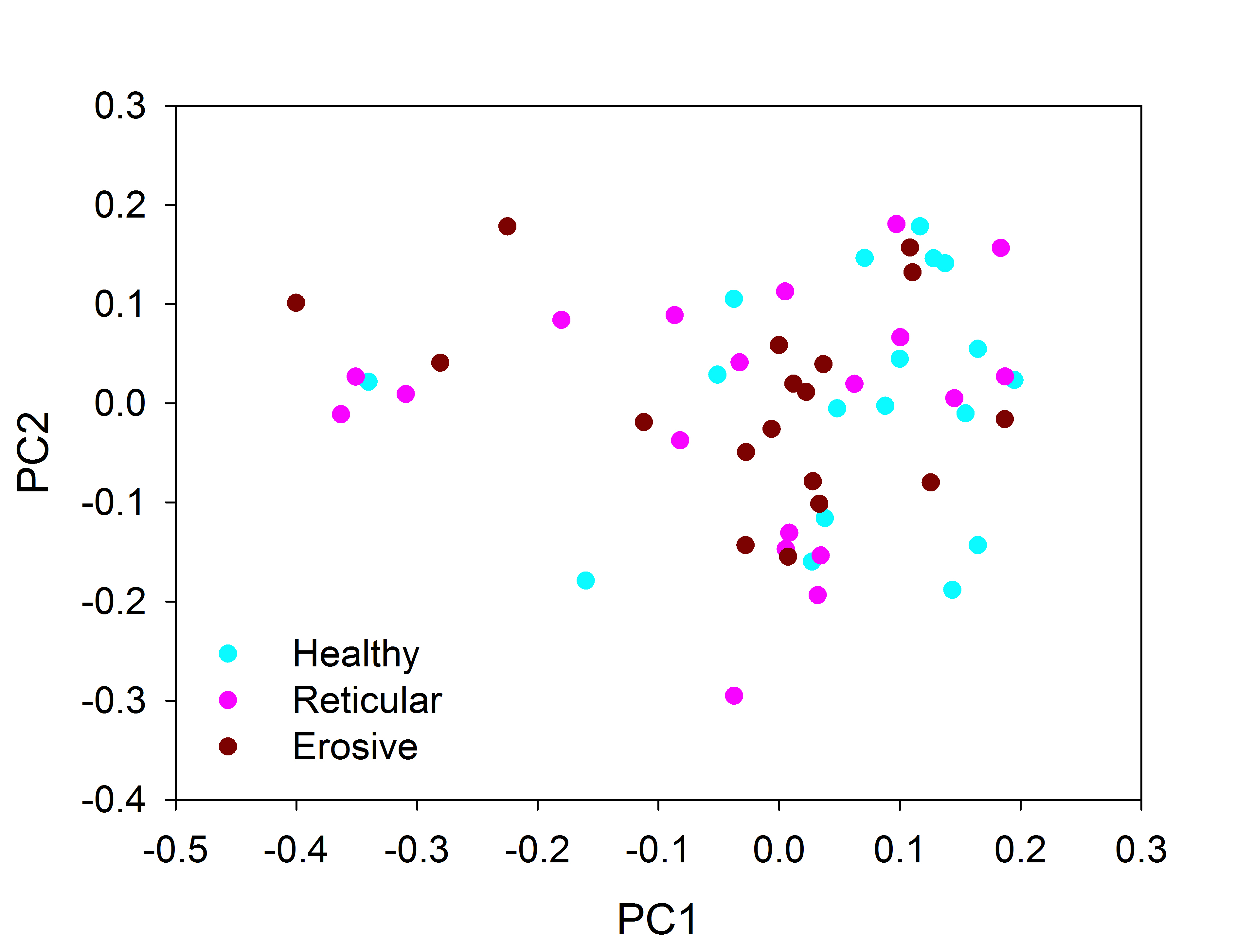
**

**Supplementary Figure S2**

**
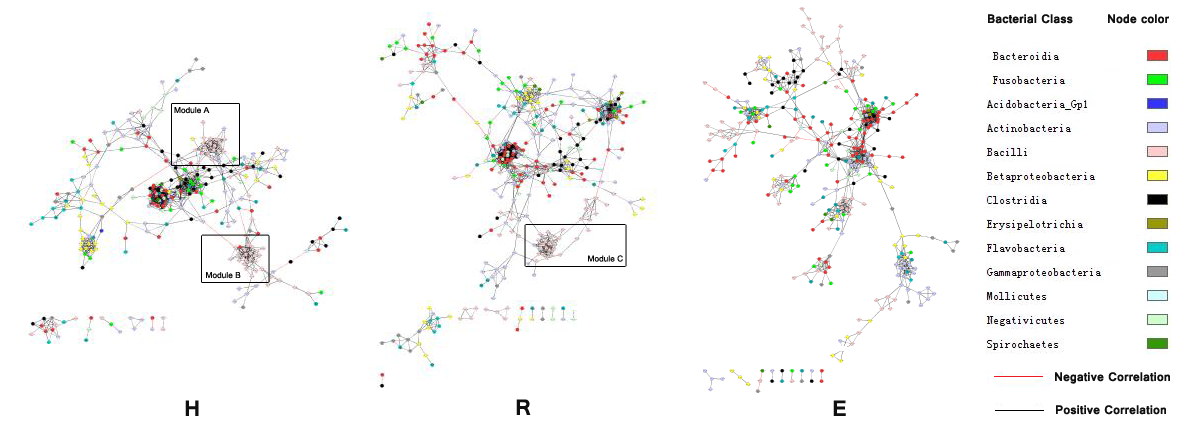
**

**Supplementary Table S1.** Summary of sequencing data.

| **Groups** | **#samples** | **#sequences per sample** | **#resampling efforts** | **#observed OTUs** | **Chao1 richness** | **Shannon index** | **Good’s coverage** |
| --- | --- | --- | --- | --- | --- | --- | --- |
| H | 18 | 27094±20420 | 12997 | 459±112 | 748±247 | 3.60±0.45 | 99.86±0.3 |
| R | 18 | 62279±12523 | 12997 | 522±102 | 834±185 | 3.81±0.46 | 99.91±0.03 |
| E | 19 | 57528±16692 | 12997 | 585±160 | 944±259 | 3.86±0.48 | 99.74±0.6 |

Note: # means “numbers of”.

**Supplementary Table S2**. Salivary levels of interleukin (IL)-17 and interleukin (IL)-23 in three experimental groups. (H, healthy control; R, reticular OLP; E, erosive OLP.)

| **Groups IL-17 (pg/ml) IL-23 (pg/ml)**  **mean±SD mean±SD** |
| --- |
| H  39.85±4.86 119.53±146  R 40.69±3.82 103.68±101.32  E 46.24±5.94 201.54±147.03 |

**Supplementary Table S3. The correlations between clinical parameters and genera. (Correlations were given as Pearson correlations.)**

| **Genera** | **Clinical scores**  **r-value P-value** | | **IL-17**  **r-value P-value** | | **IL-23**  **r-value P-value** | |
| --- | --- | --- | --- | --- | --- | --- |
| *Treponema* | 0.385 | 0.012 | 0.523 | 0.0004 | 0.236 | 0.133 |
| *Gemella* | 0.094 | 0.552 | 0.445 | 0.003 | 0.047 | 0.767 |
| *Porphyromonas* | 0.374 | 0.015 | 0.385 | 0.012 | 0.446 | 0.003 |
| *Oribacterium* | -0.320 | 0.039 | -0.383 | 0.012 | -0.183 | 0.245 |
| *Atopobium* | -0.047 | 0.767 | -0.360 | 0.019 | -0.127 | 0.421 |
| *Parvimonas* | 0.009 | 0.957 | 0.342 | 0.027 | -0.007 | 0.963 |
| *Actinomyces* | -0.162 | 0.307 | -0.330 | 0.033 | -0.256 | 0.102 |
| *Veillonella* | -0.067 | 0.675 | -0.314 | 0.043 | -0.265 | 0.089 |
| *Paraprevotella* | 0.035 | 0.827 | 0.244 | 0.120 | 0.349 | 0.023 |
| *Abiotrophia* | 0.314 | 0.043 | 0.135 | 0.396 | 0.349 | 0.023 |
| *Fusobacterium* | 0.095 | 0.549 | -0.047 | 0.769 | 0.350 | 0.023 |
